# Applications of Robotics, Artificial Intelligence, and Digital Technologies During COVID-19: A Review

**DOI:** 10.1017/dmp.2021.9

**Published:** 2021-01-08

**Authors:** Zhuo Zhao, Yangmyung Ma, Adeel Mushtaq, Abdul M. Azam Rajper, Mahmoud Shehab, Annabel Heybourne, Wenzhan Song, Hongliang Ren, Zion Tsz Ho Tse

**Affiliations:** 1 Electrical and Computer Engineering, University of Georgia, Athens, Georgia, USA; 2 Hull York Medical School, The University of York, Heslington, York, UK; 3 Department of Computer Science, University of Georgia, Athens, Georgia, USA; 4 Department of Electronic Engineering, The Chinese University of Hong Kong (CUHK), Hong Kong; 5 Department of Biomedical Engineering, National University of Singapore, Singapore; 6 Department of Electronic Engineering, The University of York, Heslington, York, UK

**Keywords:** COVID-19, Coronavirus, robotics, artificial intelligence, digital technologies

## Abstract

Many countries have enacted a quick response to the unexpected coronavirus disease 2019 (COVID-19) pandemic by using existing technologies. For example, robotics, artificial intelligence, and digital technology have been deployed in hospitals and public areas for maintaining social distancing, reducing person-to-person contact, enabling rapid diagnosis, tracking virus spread, and providing sanitation. In this study, 163 news articles and scientific reports on COVID-19-related technology adoption were screened, shortlisted, categorized by application scenario, and reviewed for functionality. Technologies related to robots, artificial intelligence, and digital technology were selected from the pool of candidates, yielding a total of 50 applications for review. Each case was analyzed for its engineering characteristics and potential impact on the COVID-19 pandemic. Finally, challenges and future directions regarding the response to this pandemic and future pandemics were summarized and discussed.

The coronavirus disease 2019 (COVID-19) pandemic has caused 6,229,408 confirmed cases and 373,973 deaths throughout the world as of June 1, 2020, based on data from the coronavirus resource center in Johns Hopkins University, and the numbers are still increasing.^1^ Many countries have quickly adopted existing technologies to respond to the disruptions caused by this pandemic,^2^ research the disease, and slow the spread of infection.^3^ These technologies mainly include robotics technologies, artificial intelligence (AI) technologies, and digital technologies, such as the Internet of Things (IoT) with next-generation telecommunication networks and big-data analytics. They are being used in sanitation, disease diagnosis, resource delivery, contact tracing, surveillance, and social control, which is a way to stop or control the movement of people, to reduce the number of COVID-19 infections and better treat patients.^2,4,5^ Furthermore, the crisis is accelerating the adoption of these technologies for the long term in many countries.^6^

In this study, technologies adopted during the COVID-19 pandemic were reviewed from 3 categories: robotics technologies, AI technologies, and digital technologies. Moreover, in each category, the technologies were reviewed and summarized based on their different applications, as shown in Table 1.^7-55^ Challenges and future directions are discussed at the end of the study.

**Table 1.** Summary of emerging technologies during the COVID-19 pandemic

	Application	Principle	Authors/organizations	References
Robotics	Sanitation in public areas	Robotic vacuum	SoftBank Robotics	[7, 8]
		Disinfection robot	Nanyang Technological University	[9]
		Tank-style, remote control disinfecting robot	NA	[10]
		Portable hand sanitizer dispenser robot	Zhen Rhobotics Corp	[11]
	Sanitation in hospitals	Aerosol disinfection robot	Shanghai TMiRob Technology	[12]
		UV-C light disinfection robot	UVD Robots	[13]
		Intelligent disinfection robot	TMiRob	[14]
		UV light robot	Xenex Disinfection Services	[15]
		Autonomous cleaning robot	Seoul National University Hospital (SNUH)	[16]
	Delivery inside hospitals	Autonomous service delivery robots	Pudu Technology	[17, 18]
	Delivery outside hospitals	Larger autonomous delivery robot, robotaxi	JD Logistics	[19]
		Large unmanned distribution vehicles	White Rhino Auto Company	[20]
		Large autonomous delivery, self-driving	Meituan	[21]
		Small, self-driving delivery cart	ZhenRobotics Corp	[22]
	Patrolling	Small autonomous robot	NA	[23]
		Small, semi-autonomous patrol robot	Boston Dynamics – Owned by Softbank	[24]
		Autonomous, self-driving, patrol robot	Developed by Guangzhou Gosuncn Robot Company	[25]
	Screening	AI-powered, screening robot, video-conferencing	Robotemi	[26, 27]
		AIMBOT: Autonomous, AI-powered indoor monitoring robot, mask recognition Cruzr: AI-powered medical service robot, video call	Ubtech	[28]
		Autonomous screening robots	Ubtech	[29]
		LIDAR and IR/optical cameras for screening, 4G connection	Ubtech	[30]
AI technologies	Health-Care Diagnosis	AI-assisted analysis for COVID-19 CT images	Huawei, Huiying Medical, SenseTime, Ping An Smart Healthcare, Beijing Infervision Technology Co. Ltd, Qure.ai, Shanghai Public Health Clinical Center (SPHCC) And Yitu, Thailand’s Ministry of Digital Economy and Society	[31-37]
		AI-powered genetic analysis of suspected COVID-19 cases	Alibaba	[38]
	Health-Care Information tool for a doctor	“Bot MD” – awareness of changing information relating to COVID-19	National University Hospital (NUH) and Tan Tock Seng Hospital (TTSH)	[39]
	Health-Care health consulting	“Huya Live” – AI doctor to provide personal hygiene advice to the public on live-streaming video for one week	SenseTime	[40]
	Monitoring/detection in public areas-screening	Remote temperature detection (accuracy ±0.3°C) Scan up to 10 people/second	SenseTime	[41]
		Remote temperature detection	Megvii Technology Limited	[42]
	Monitoring/detection in public areas-transportation	“Smart Commute System” – paying at subway using QR code & face scanning	SenseTime	[43]
Digital Technologies	Tracking	Geofencing technology, Compulsory quarantine monitored by an app	Hong Kong government	[44, 45]
		Digital contact tracing based on Bluetooth	SGUnited, GovTech, Ministry of Health Singapore	[46, 47]
		GPS tracking app and CCTV footage	South Korean government	[48]
		Mapping and notification of COVID-19 cases	Various developers in South Korea	[49]
		Digital QR health codes	Chinese government	[50]
	Health care	Remote 5G technology	ZTE	[51]
		Cloud-based Honghu Hybrid System	Southern Medical University	[52]
	Daily life	Pharmacy inventory apps	South Korean government	[53]
		Online VR house viewing	Evergrande Sunshine Peninsula, Vanke	[54, 55]

## Methods

The keywords “COVID-19 technology,” “COVID-19 robotics,” “COVID-19 AI technology,” and “COVID-19 digital technology” were used in a Google search to identify news articles and scientific reports for review. The results were then cross-checked with Bing search and Yahoo search to ensure no relevant news articles/scientific reports were missed. The initial selection was based on the titles of the news articles/scientific reports, and 163 candidates were identified.

**Figure 1.**
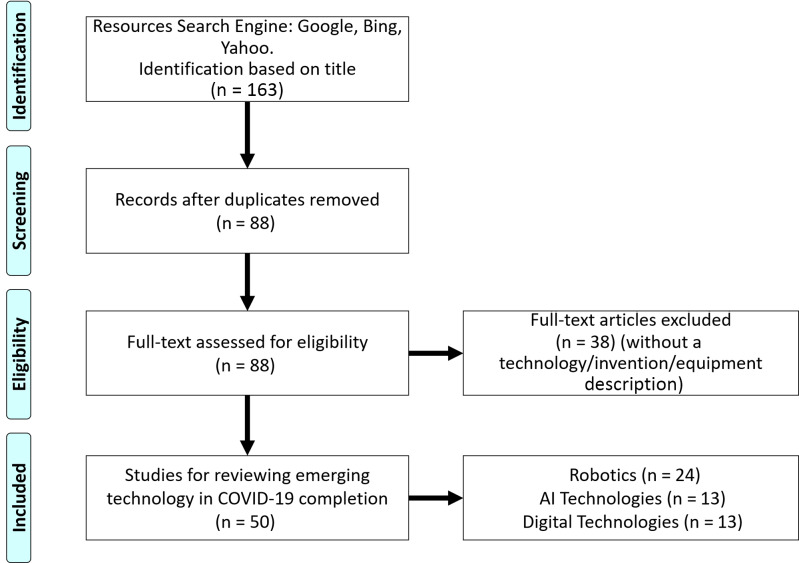
The research process for each category of literature addressed in this article.

After the initial candidates were selected, they were subjected to elimination evaluations. First, duplicate or similar news articles/scientific reports were eliminated, and the number of candidates was reduced to 88. Second, each news article/scientific report was read in full and manually analyzed to remove any without a technology/invention/equipment description, resulting in a pool of 50 candidates. These 50 candidates were then divided into 3 categories: robotics, AI technology, and digital technology. Under these 3 categories, the candidates were further divided into different sections based on their functions and reviewed in depth (Figure 1).

## Results

### Robotics Technologies

A dichotomy currently exists between the requirement for the minimization of human contact to reduce infection transmission rates and the need for humans to carry out the essential tasks of their daily lives. A surge in the creation of robotic technologies has been observed to bridge this gap, including robots designed for sanitation, delivery, patrolling, and screening that aim to work alongside humans in efficiently reducing the burden of the pandemic while maintaining the quality of life. Whether placed in areas of high infection risk, like hospitals, or in public areas, the possible applications of robotic technology both during this pandemic and in the future seem infinite. Therefore, a review of currently available robotic technologies is essential for the further development and widespread use of robots to fight the pandemic.

#### Sanitation

The importance of adopting strict sanitary habits has never been more important, because the main transmission route for COVID-19 is by means of respiratory droplets and direct contact, and droplets may land on surfaces where the virus can remain viable. Thus, the immediate environment of an infected individual can act as a source of transmission,^56^ which highlights the need for sanitation methods that can be carried out in accordance with social distancing rules while also ensuring the safety of all individuals within an infected population.

**Figure 2.**
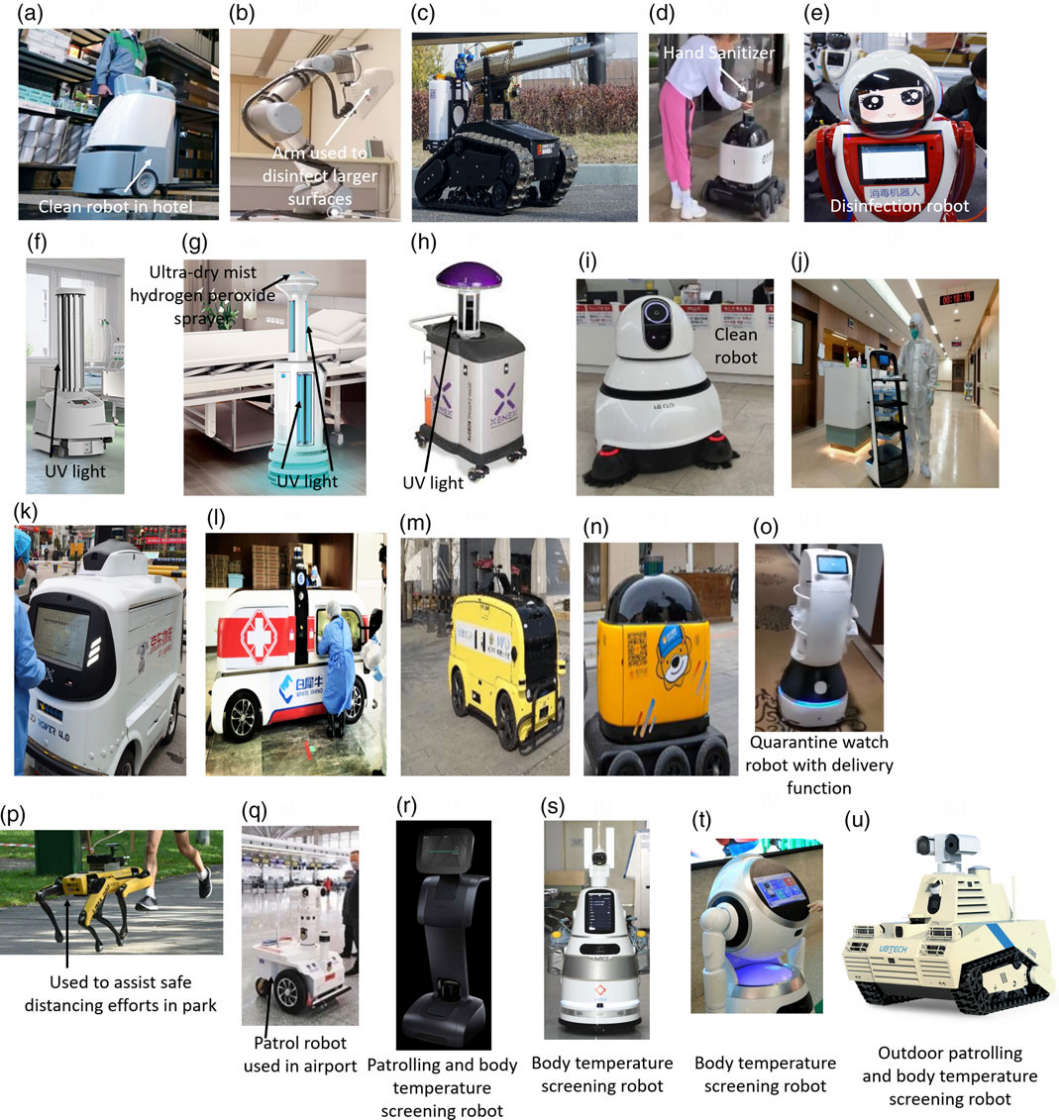
(a) Cleaning Robot from SoftBank Robotics.^8^ (b) XDBot by Nanyang Technological University.^9^ (c) Tank-style, remote-controlled disinfecting robot.^10^ (d) Portable hand sanitizer robot from Zhen Rhobotics Corp.^11^ (e) Aerosol Disinfection Robot from Shanghai TMiRob Technology.^12^ (f) UV-C light Disinfection Robot by UVD Robots.^13^ (g) Intelligent Disinfection Robot by TMiRob.^14^ (h) UV light robot by Xenex Disinfection Services.^15^ (i) Autonomous Cleaning Robot by Seoul National University Hospital.^16^ (j) Pudu Technology’s autonomous service delivery robot.^18^ (k) JD Logistics’ large self-driving delivery vehicle.^19^ (l) White Rhino Auto Company’s large self-driving delivery vehicle.^20^ (m) Meituan’s large self-driving delivery vehicle.^21^ (n) ZhenRobotics’ RoboPony.^22^ (o) Quarantine watch robot.^23^ (p) Boston Dynamics’ Spot being used in Bishan-Ang Mo Kio Park, Singapore.^24^ (q) Smart patrol robot being used in Guiyang Airport, China.^25^ (r) Temi robots,^26^ (s) AIMBOT, and (t) Cruzr robots from UBTech.^28^ (u) Atris outdoor screening and patrolling robot from UBTech.^30^

Companies and universities have started to use robotics to meet the stringent sanitary requirements for hospitals and communal public areas to be safe to enter. One example is the use of the robotic vacuum Whiz, developed by SoftBank Robotics (Figure 2a), in Tokyo hotels housing COVID-19 patients with mild symptoms.^8^ Whiz can be used in open settings due to the on-board BrainOS*,^1^ the integrated AI system, which determines the best route through the surrounding environment and avoids obstacles such as stairs and human movement.^57^ Whiz can also be remotely monitored by cleaning staff from a smartphone or PC.

Nanyang Technological University has developed XDBot (Figure 2b),^9^ a disinfection robot controlled with a laptop or tablet. XDBot uses an electrostatically charged nozzle to spray positively charged chemicals onto negatively charged surfaces for highly effective cleaning.^58^ Furthermore, XDBot has a 6-axis arm, which allows it to have a greater reach compared with other robots. This enables it to spray areas thought to be unreachable, such as light switches and doorknobs.^58^ Coupling this with an 8.5-L tank and 4-h run time, XDBot can outcompete human cleaning capacity, which makes it a useful tool for sanitizing indoor public areas. Efforts have also been made to use similar spray technologies to sanitize larger areas in a shorter amount of time. These include tank-style robots (Figure 2c) that are able to climb stairs much more easily than wheeled robots with similar technologies.^10^ These robots are also controlled by remote controls, making them easy to operate.^10^

While some robots have been designed to vacuum floors and others to spray disinfectant, Zhen Rhobotics Corp. has designed a portable hand sanitizing robot (Figure 2d). This robot can automatically navigate through outdoor areas and deliver hand sanitizer to individuals.^11^ Robotics such as these will be very useful when attempting to ensure a high number of individuals in a population have access to sanitary products, potentially reducing the spread of COVID-19.

In addition to public areas, sanitation robots are also extremely useful in hospital settings to reduce the risk of transmission from COVID-19 patients to other patients and hospital staff. Shanghai TMiRob Technology is 1 of many companies answering this call; they have developed a robot (Figure 2e) that combines chloric acid and plasma to disinfect environments in which humans and robots are present.^12^ The robot can move automatically to areas frequently visited by patients and medical staff, therefore, minimizing people’s exposure to infected surfaces.

UVD Robots has also adopted self-driving capabilities in its UV-Robot (Figure 2f), which is composed of UV-C light emitting bulbs.^13^ The machine aims to prevent and reduce the spread of infectious diseases, viral and bacterial, and other types of harmful organic microorganisms by breaking down their DNA structure.^59^ The UV-Robot has been designed to be used with minimal input from cleaning staff, thus making it very user friendly. Some of its useful features include control over the robot by means of an app and a security checklist before the application of UV-C lights to ensure safety.

Similar technologies are being used in the Intelligent Disinfection Robot designed by TMiRob (Figure 2g)^14^ and the LightStrike Robot designed by Xenex Disinfection Services (Figure 2h).^15^ The Intelligent Disinfection Robot acts as a carrier to integrate 3 disinfection modules: ultraviolet rays, ultra-dry mist hydrogen peroxide, and plasma air filtration.^60^ This makes the robot suitable for cleaning both air and object surfaces in hospitals, and it requires little human intervention due to its autonomous navigation technology. Likewise, the LightStrike Robot emits a wavelength between 200 and 312 nm, which includes both UV-B (280-315 nm) and UV-C (200-280 nm), making it unique to other ultraviolet emitting technologies.

In an alternative approach, LG CLoi CleanBot (Figure 2i) was designed to prevent secondary COVID-19 infections in hospitals.^16^ Created by a collaborative effort between Seoul National University Hospital and LG Electronics, the robot features indoor autonomous driving, obstacle-avoidance technology, and an H13 high-efficiency particulate air filter to improve air quality.^16^ This technology is still in the early stages of development, but once the technology is fully developed, it aims to reduce the risk of in-hospital infections by eliminating human-to-human contact while substantially increasing work efficiency.

#### Delivery

The importance of contactless delivery is shifting from convenience to necessity during the pandemic due to the need for minimizing physical contact. With delivery volumes across all industries having grown by 77% and grocery delivery by 80%,^61^ it is evident that people are opting for safer shopping methods. To meet this demand, several technologies have been created for deliveries both in hospitals and the general public in the effort to reduce the burden of the pandemic.

In areas of high infection risk, such as hospitals, robotic delivery is required to decrease the workload of health-care workers and reduce the spread of COVID-19. Applications include delivery of medication, food, documents, and infectious samples for testing. As shown in Figure 2j, autonomous service delivery robots by Pudu Technology are currently being used in Wuhan to deliver cooked foods and medications to people being kept in quarantine.^17^ Equipped with all-terrain 3D mapping, large-scale visual and sensor navigation technologies,^18^ and shelves designed to hold multiple items at once, these robots are able to navigate efficiently to make deliveries to many patients. However, with this design, there is a risk of items being touched by multiple people, which increases the risk of cross-infection.

The technologies used for delivery in public areas can be divided into 2 types: larger vehicles and smaller vehicles. The larger automated vehicles, currently made by JD Logistics, White Rhino Auto Company, and Meituan (Figures 2k, l, and m, respectively), are designed to deliver large volumes of daily necessities and medical supplies to hospitals and residential compounds for both medical personnel and general customers.^19^ As the demand for online shopping grows due to lockdowns, these vehicles provide an efficient way to prevent the spread of disease while meeting basic needs, which improves the quality of life. The self-driving vehicles now travel on the public roads of China due to the restriction of traffic during the pandemic.^20^ With the ability to travel 100 km and carry up to 100 kg of goods, several deliveries can be made per trip.^21^ Highly technological sensors are integrated into the design to reduce delivery dangers and interruptions such as weather conditions and to enable the vehicles to safely avoid obstacles and identify traffic lights and other road signs before parking at the recipient’s door. The whole delivery process is monitored by security officers to ensure the completion of delivery. Advanced AI algorithms are used in the vehicles to upgrade software engineering, and laser radar is used to enhance connectivity and allow the vehicle to adjust to real logistics scenarios, such as adjusting speed according to real road conditions.^19,20^ However, despite their efficiency, larger self-driving vehicles remain costly, and the possibility of cross-infection is a concern due to their ability to make several deliveries.

Smaller vehicles, such as ZhenRobotics’ Robopony (Figure 2n), travel in nonmotorized areas and are used as “last-mile delivery.” They are designed to deliver smaller volumes of food and groceries to allow people to stay home. One advantage is the reduced risk of cross-infection, because the vehicles are only able to deliver a few items to 1 customer and are easily cleaned. Due to its small size, it is pedestrian-friendly, and by being colorful, it attracts attention from bystanders to prevent any disturbance in delivery. Special sensors have been fitted to react to lights and obstacles while being able to signal to cars when the vehicle is entering a crosswalk. Other advantages include the ability to patrol by identifying customers with bare faces in public and reminding them to put on a mask while broadcasting information about safe practices to further reduce the risk of infection.^22^ Despite their growing demand, smaller vehicles are not able to travel far and so in some cases are used as “last-mile delivery,” meaning that they have to be loaded in a car and driven to a nearby location before being taken out and operated for delivery. This raises significant problems of infection risk as it involves human interaction. The vehicles also pose a small safety risk to pedestrians, especially for the elderly and disabled, who are less mobile and may be unable to avoid a vehicle in the event its obstacle avoidance sensors malfunction. Issues could also arise if these vehicles are purposefully tampered with during delivery.

#### Patrolling and Screening

Social restriction policies have been used to effectively curb the spread of COVID-19 from asymptomatic carriers. As countries aim for exit strategies from lockdown to return to economic growth, the second wave of infections has presented as a possibility.^62^ To avoid this problem, many robotics and technology companies worldwide have been developing or repurposing existing robotic technology to enforce and facilitate social distancing policies.^63^ In addition, to restrict the spread of COVID-19 from carriers to others, early detection by means of screening is critical so that isolation protocols can be put in place. This is another area where robotics are beneficial, as they can be designed to administer screening tests instead of medical personnel.

A novel implementation of robotic technology to enable social distancing in high-risk areas is the use of small, autonomous robots (Figure 2o) to patrol hotels, such as Beijing’s “quarantine hotels,” to monitor the quarantine situation and deliver food, bottled water, and other packages to those self-isolating. These robots are equipped with a speaker to communicate with residents and a high-resolution camera array to recognize their surroundings and navigate through hotel hallways,^23^ eliminating the need for human foot traffic and, thus, lowering the potential spread of the virus.

Robots have also shown potential in enforcing social distancing policies in public areas, protecting key workers from potentially exposing themselves to the virus. Singapore is currently trialing the use of Spot, a dog-like robot shown in Figure 2p developed by Boston Dynamics, to help the volunteer Safe Distance Ambassadors notify the public when safe distances are not being maintained. Currently, the operation of Spot is semi-autonomous, where the main control is given to an operator, but the robot can detect obstacles and avoid them.^24^

Newer generation AI robots have allowed for autonomous functions and subsequent reduction in virus exposure and labor cost. One example is a line of robots designed by Guangzhou Gosuncn Robot Company initially intended to be used in policing, but that has been upgraded with multiple infrared (IR) cameras and high-resolution cameras to screen the body temperature of up to 10 people at once in a 5 meter radius and detect whether individuals are wearing a face mask (Figure 2q). The robots also have cloud-based 5G functionality, allowing them to be part of an IoT that facilitates communication between robots on duty.^25^

Another device is Temi (Figure 2r), a personal, AI-powered, self-navigating robot modified to be deployed in entrances of busy or high-risk areas, such as hospitals. It has built-in IR cameras and a thermometer to detect those suffering from pyrexia, as well as visible spectrum cameras that can determine whether masks are being worn. It also has video calling capabilities using the optical camera, meaning that it can be used in hospitals for remote consultations between doctor and patient. Furthermore, it can be equipped with a sink to promote hand hygiene.^26,27^ AIMBOT and Cruzr, 2 of the 3 anti-epidemic robots made by Ubtech (Figure 2s and t), share many features with Temi, including IR and optical cameras for temperature screening and mask recognition and AI software for autonomous operation.^28^ Cruzr can perform an initial analysis of symptoms for diagnostic purposes and enable live consultation with health-care staff. It can also review inpatients on hospital wards by analyzing vital signs and alerting health-care staff of any abnormal results. This makes it better equipped for use in hospitals. However, the use of touchscreen LCD panels imposes significant cross-contamination risks, as several studies have shown that different strains of coronavirus can survive on dry surfaces for up to 5 d.^64^

Atris (Figure 2u) is the outdoor screening and patrolling robot that is a member of the anti-epidemic robots from Ubtech. It has a comparable array of sensors, such as LIDAR and IR/optical cameras, for screening. It also has a rugged design, optimizing it for outdoor use in public areas where rough ground and harsh weather could damage more delicate models. However, it can only support 4G connectivity. This is not an issue in more rural communities that have not been equipped with 5G, but it is a limiting factor when attempting to stream large amounts of data to a central command center, such as during a situation requiring further intervention by an operator.^29,30^

### AI Technologies

AI has the potential to support health care in a novel capacity during the COVID-19 pandemic. Thus far, AI has been demonstrated to be useful for analyzing computed tomography (CT) scans, keeping doctors up to date with the changing information surrounding COVID-19, engaging in health promotion, and even detecting people with certain symptoms of COVID-19 from afar. With this combination of functions, AI has improved the efficiency of health-care workers significantly and reduced diagnosis time.

#### Health Care

CT exam has played a key role in diagnosing COVID-19 cases quickly because the standard method reverse transcription polymerase chain reaction (RT-PCR) is much slower. However, it still takes radiologists a long time to analyze CT images, resulting in a huge workload for radiologists considering the number of potential COVID-19 cases. According to various reports, Huawei,^31^ Huiying Medical,^32^ SenseTime,^33^ Beijing Infervision Technology,^34^ Ping An Smart Healthcare,^34^ Quei.ai,^35^ Yitu,^36^ and Thailand’s Ministry of Digital Economy and Society^37^ have developed similar AI systems that can analyze CT images taken from COVID-19 patients. The technology is claimed to be able to interpret a CT scan with over 90% accuracy within 15 s.^34^ This significantly reduces the diagnosis time and workload for radiologists. However, the application of AI for COVID-19 diagnosis does not stop at CT scan analysis. For example, Alibaba developed an AI-powered system that conducts a genetic analysis of suspected COVID-19 cases.^38^

COVID-19 is not thoroughly understood, as information on the disease is constantly changing and updating. As such, the National University Hospital (NUH) and Tan Tock Seng Hospital (TTSH) developed “Bot MD,” an AI toolkit that collects evidence-based clinical information from medical associations, the Ministry of Health of Singapore, and volunteer doctors to provide frontline doctors with the most up-to-date information regarding COVID-19 and its management. Bot MD is also able to provide information on the hospital’s latest operational procedures, the doctors on call, and any guidelines.^39^ TTSH is also using AI in its monitoring of hospital operations. The tool “Command, Control and Communications (C3)” tracks patients in their journey through health care, thus allowing the system to proactively determine likely situations before they occur, allowing for better distribution of resources.

**Figure 3.**
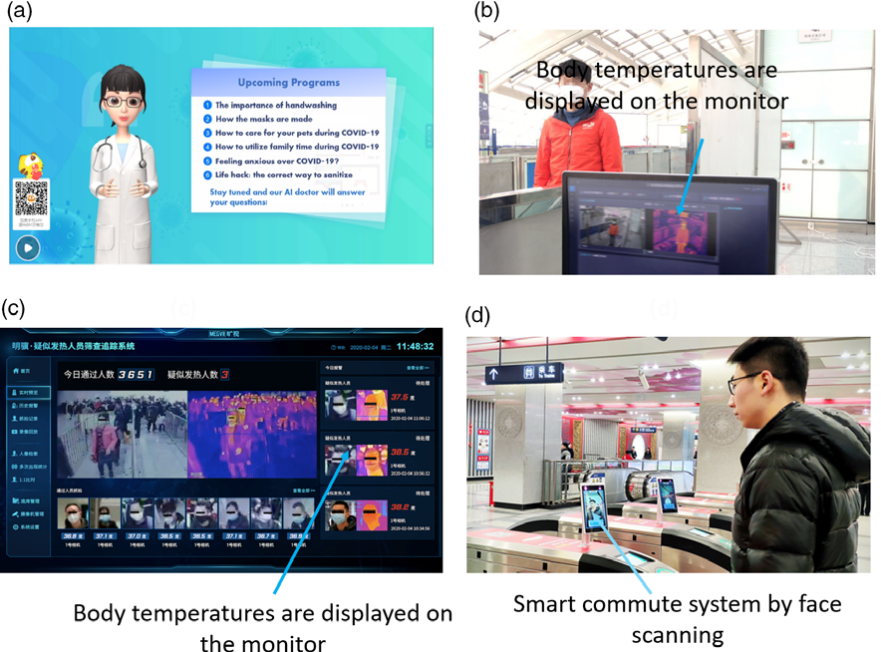
(a) AI doctor from SenseTime to provide personal hygiene advice on a live-streaming video platform.^40^ AI powered screening system from (b) SenseTime^41^ and (c) Megvii.^42^ (d) SenseMeteor Smart Commute system from SenseTime.^43^

Also, with resources spread thin due to COVID-19, doctors are unable to continue 1 of their core duties: health promotion. Therefore, SenseTime developed “Huya Live,” illustrated in Figure 3a. This AI doctor engaged with the general public on different health themes for 1 wk. Each day varied in topic, from managing mental health to how to wear a mask. Viewers were able to interact with the AI by asking questions and receiving responses, with facial expressions and gestures to match the vocals.^40^

#### Detection of Potential Cases in Public Area

In addition to providing help for health-care workers, AI has also shown promise in detecting potential COVID-19 cases in public. One method is by using an AI-powered system integrated with infrared technology that screens an individual’s temperature, because fever is a common symptom of the disease.^65^ SenseTime (Figure 3b) and Megvii Technology Limited (Figure 3c) have both developed this technology with an accuracy of ± 0.3°C, and both of them can screen up to 10 or 15 people/s.^41,42^ These devices have been deployed at the entrances and exits of public places that regularly have large streams of people, such as train stations, airports, and shopping malls, to detect potential cases. Upon detection of an individual with a fever, the system automatically alerts a staff member who can then confirm the temperature with a manual check. Even in situations where people are wearing hats and face coverings, these systems can still work properly.

Furthermore, SenseTime has also adopted a system called SenseMeteor Smart Commute to reduce interpersonal contact during the pandemic. This tool has been implemented in China’s public subway system.^43^ As shown in Figure 3d, the system uses a combination of face scanning and QR codes as the new payment method. This system is easily installed on existing physical ticket gates, and customers are not required to install any additional applications as it has been integrated into the current metro apps.

### Digital Technologies

The use of big data has enabled worldwide organizations to forecast the outbreak and has been used to support public health decisions. Public health organizations have partnered with leading social media networks to address the public, provide updates, and combat rumors.^4^ AI algorithms are being developed as methods of screening the population, as well as acting as a ‘bot’ which can respond to patients, educating them on symptom recognition and public health measures.^4^

Previous outbreaks have demonstrated that early data sharing, mobile tracking, and quarantining measures are effective strategies.^3^ There are, of course, several barriers to digital technologies, including patient acceptance, government support, health insurance providers, investors, and regulation.^6^ Emerging digital technology has been described as limited in terms of quality and personal security testing. Ransomware, spyware, and malware programs have masked themselves as COVID-19 tracking apps.^6^

#### Tracking

Mapping out the locations and contacts of COVID-19 patients during the proposed infectious period could help identify the transmission of the virus and notify individuals who may be at risk of contracting the disease.^6^ Contact tracing has moved, in part, from a human to a digital process.^66^ As well as getting COVID-19 cases under control, digital contact tracing could be vital in the next phase of the pandemic; for example, to introduce targeted quarantine rather than keeping an entire country in quarantine.^66^

**Figure 4.**
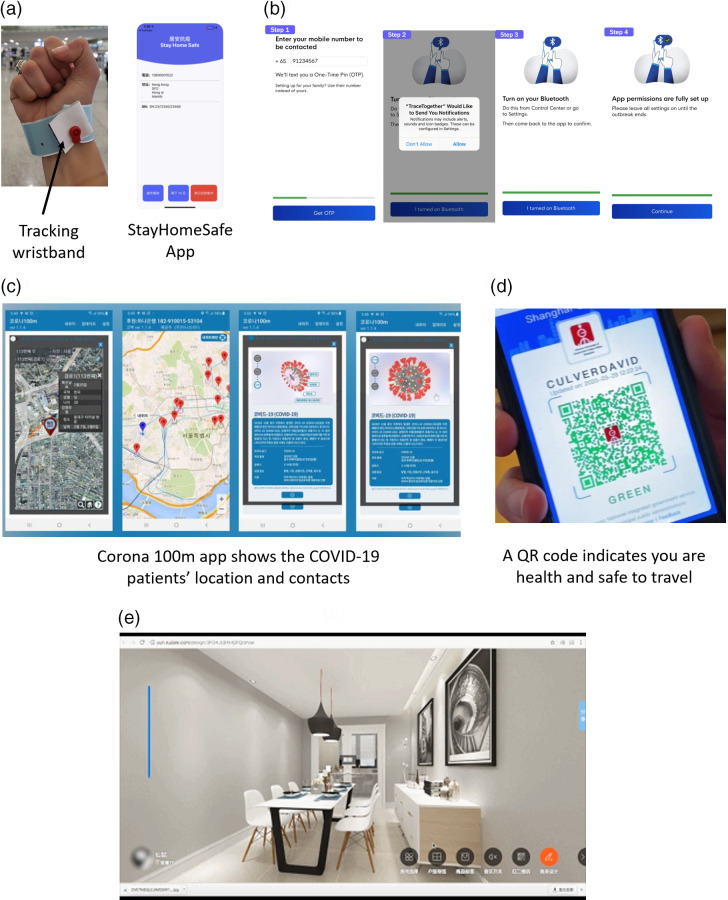
(a) Tracking wristband and app from theHong Kong government.^45^ (b) TraceTogether app from the Singapore government for tracking potential COVID-19 patients.^47^ (c) Corona 100m app used for tracking COVID-19 patients in South Korea.^49^ (d) The QR code used in China to track potential COVID-19 patients.^50^ (e) Online VR view of a new house for customers.^54^

Although countries including Singapore, South Korea, and China have used technology alongside public health measures, the FluPhone, which was launched in 2011, marked the first use of a mobile phone app to record (anonymous) data on how often individuals are in close contact with each other through Bluetooth, GPS, and self-reporting systems.^67^ Hong Kong introduced the StayHomeSafe app to ask everyone arriving in the area to receive a wristband, which is subsequently paired with an app to create a digital signature of an individual’s home (Figure 4a).^44,45^ If found to be leaving the house, an individual can face prosecution.^44^ Similarly, TraceTogether was launched in Singapore, as shown in Figure 4b.^46^ When users with the app installed are near each other, Bluetooth signals are exchanged, and the encounters are encrypted and stored in the phone for 21 days.^47^ These data can be decrypted and used if a user is subsequently diagnosed with COVID-19.^47^ However, 1 mo after launching, only 12% of people in Singapore had downloaded the app.^66^ South Korea moved in a similar direction, using GPS, bank records, and closed-captioning television (CCTV) footage to trace the movements of COVID-19 positive patients, releasing the information online for 2 wk.^48,66^ Apps such as Corona 100m (Figure 4c), Corona Map, and Close Contact Detector have attempted to make this information more visual.^49^

An idea unique to China is the introduction of health QR codes as shown in Figure 4d. The codes are linked to widely used platforms, including Alipay, Tencent, and WeChat. Individuals enter their personal details and their travel and symptom history to generate 1 of 3 health codes. Red indicates individuals must quarantine for 14 days; amber indicates individuals must quarantine for 7 days; and green indicates individuals are free to go wherever. Initially, different cities and provinces developed their own versions of the health code; however, this caused issues for travelling because not all cities recognized each other’s QR codes.^50^ In some provinces, the health codes have been removed already, but in others the codes govern where people can go.^50^

#### Health Care

Tracking aside, the COVID-19 pandemic has necessitated other developments in health-care technology as well. ZTE, a Chinese telecom provider, converted a conference room in a Chinese hospital into a remote 5G diagnosis and treatment system. The technology has since been launched in 30-50 Chinese cities, with the hope of implementing 5G in all cities by the end of 2020.^51^ In addition, Hitachi Vantara, BurstIQ, and the American Heart Association launched the COVID-19 data challenge in an attempt to attract researchers to analyze datasets and assess relationships among COVID-19 mortality and ethnicity, gender, geography, and income. There are 2 stages to the challenge, with monetary rewards at the end of each.^51^

A pilot system set up in just 72 h by a team of 40 experts following the outbreak of COVID-19 in Wuhan was launched in Honghu. The Honghu Hybrid System (HHS) combined case report systems, electronic medical records, and social media platforms (eg, WeChat) to provide real-time data. Over 95% of the population were surveyed, and information of symptoms, psychological status, contact history, social behavior, and physical condition (eg, muscle soreness or not, feel tired or not) were collected. The data inferred public health measures enabled the analysis of close contact history and monitored local outbreaks. Despite this rapid transformation, it was only trialed in 1 city, and there were reports that the system was not user friendly.^52^

#### Daily Life

Commerce platforms such as Alibaba, Amazon, McDonald’s, and Starbucks have introduced no-contact purchase and delivery in an attempt to reduce business disruption amidst the pandemic.^68^ If citizens choose to venture out in public, MaskGoWhere provides information on where to obtain a face mask.^69^ Moreover, to discourage unnecessary queues at pharmacies and address complaints about face mask shortages, the South Korean government published data on face mask sales. It also issued a plea to the private app-developing sector to come up with an app to keep the public up to date with the inventory statuses of pharmacies across the country. Around 22,000 of 23,000 pharmacies in the country have agreed to share their data.^53^

Moving away from the health sector altogether, real estate companies have begun encouraging virtual reality (VR) house viewings. The VR viewing channel launched by Evergrande Sunshine Peninsula features panoramic displays of houses, including the outside environment, and an individual property consultant is available 24 h a day to answer any queries (Figure 4e).^54^ Evergrande reportedly sold 58 billion yuan in real estate within 3 d of opening. Since February, the frequency of VR viewing has risen dramatically.^55^

## Discussion

Robotics, AI, and digital technologies have provided major assistance during the COVID-19 pandemic and shown their promising future in society. However, challenges remain for these technologies to be applied widely and integrated into a long-term shift in lifestyle.

Cost is the biggest challenge for these technologies, particularly robotics, to be implemented widely. Although robots have great potential as tools to meet various needs in clinical and daily life, the high cost of robots means they will most likely be accessible only to the wealthiest hospitals and cities, where only a small fraction of people can benefit. Another concern with robots is safety. Many delivery robots have been implemented in hospitals and public areas during this pandemic to reduce person-to-person contact, and they can be navigated to their destinations automatically with AI or other technologies. However, the safety of these delivery robots is still questionable, especially in hospitals. Considering the small aisle room in hospitals, any increase of people and transportation in hospitals will increase the difficulty of robot navigation.

Security and privacy is another concern, because some countries may track the spread of COVID-19 by monitoring people’s daily activities through AI-driven facial recognition or smartphone apps.^70-73^ These data should be used under strict guidance due to the private information included. In addition to the supervision of the government, other methods should be applied to protect privacy, such as restricting the data use period and enforcing data protection rights, including the right to delete data and the use methods of the data.^73,74^

Finally, some lessons can be learned from the COVID-19 pandemic. Most of the technologies used in the pandemic have been adapted from pre-existing technologies. Although research laboratories and startups are creating new technologies and prototypes during the pandemic to help health-care workers and the public, their products may not reach the market in time, or they may be unable to grow their customer base fast enough to reach a critical mass. Overall, it is more efficient and scalable to repurpose existing technologies than invent new ones during a pandemic/crisis.^75^ To promote this approach, governments should encourage companies and research groups to explore the potential applications of existing technology/products. Moreover, governments should invest money to do technical reserves in nonpandemic/crisis periods so that these technologies can be commercialized as helpful products during future pandemics/crisis periods.

## Conclusion

Many challenges in the management of COVID-19 for both public health agencies and hospitals have been caused by the unexpectedly large and widespread impact of the pandemic. Maintaining social distancing, reducing person-to-person contact, achieving rapid diagnosis, tracking virus spread, and providing sanitation are some of the major challenges in the pandemic period. Many countries, especially East Asia countries, have presented a faster and more efficient response to these challenges by adopting existing technologies in hospitals and public areas. In this review study, existing technologies adapted for fighting COVID-19 were considered for review. Ultimately, 50 technologies in the areas of robotics, AI, and digital technology were selected for review. These technologies have been implemented in sanitation for hospitals and public areas, delivery in hospitals and public spaces, patrolling, screening, diagnosis, health consulting, and virus tracking. The engineering characteristics of each invention were summarized, and the potential to make a significant impact on the pandemic response was evaluated and discussed. Some of these technologies are still unlikely to be deployed widely due to their high cost, and problems related to security and privacy should also be considered when deploying these technologies. To better respond to pandemics in the future, lessons can be learned from the COVID-19 pandemic. We may be able to develop protocols for how to adapt pre-existing technologies to meet the needs of future pandemics or other crises more efficiently and on a larger scale. Additionally, speeding up the development and deployment of new technologies after the pandemic will create a bigger pool of pre-existing technology.
